# Inhibition of Oxidative Stress and Lipid Peroxidation by Anthocyanins from Defatted *Canarium odontophyllum* Pericarp and Peel Using *In Vitro* Bioassays

**DOI:** 10.1371/journal.pone.0081447

**Published:** 2014-01-09

**Authors:** Hock Eng Khoo, Azrina Azlan, Amin Ismail, Faridah Abas, Muhajir Hamid

**Affiliations:** 1 Department of Nutrition and Dietetics, Faculty of Medicine and Health Sciences, Universiti Putra Malaysia, Selangor, Malaysia; 2 Laboratory of Halal Science Research, Halal Product Research Institute, Universiti Putra Malaysia, Selangor, Malaysia; 3 Department of Food Science, Faculty of Food Science and Technology, Universiti Putra Malaysia, Selangor, Malaysia; 4 Department of Microbiology, Faculty of Biotechnology and Biomolecular Sciences, Universiti Putra Malaysia, Selangor, Malaysia; University of Sassari, Italy

## Abstract

*Canarium odontophyllum*, also known as CO, is a highly nutritious fruit. Defatted parts of CO fruit are potent sources of nutraceutical. This study aimed to determine oxidative stress and lipid peroxidation effects of defatted CO pericarp and peel extracts using *in vitro* bioassays. Cell cytotoxic effect of the CO pericarp and peel extracts were also evaluated using HUVEC and Chang liver cell lines. The crude extracts of defatted CO peel and pericarp showed cytoprotective effects in *t*-BHP and 40% methanol-induced cell death. The crude extracts also showed no toxic effect to Chang liver cell line. Using CD36 ELISA, NAD^+^ and LDL inhibition assays, inhibition of oxidative stress were found higher in the crude extract of defatted CO peel compared to the pericarp extract. Hemoglobin and LDL oxidation assays revealed both crude extracts had significantly reduced lipid peroxidation as compared to control. TBARS values among defatted CO pericarp, peel, and cyanidin-3-glucoside showed no significant differences for hemoglobin and LDL oxidation assays. The protective effects of defatted CO parts, especially its peel is related to the presence of high anthocyanin that potentially offers as a pharmaceutical ingredient for cardioprotection.

## Introduction

Antioxidant is a substance able to scavenge free radicals and reduce oxidative stress. It is naturally and abundantly found in plants. The major antioxidant discovered in plants is phenolic compound [1]. Phenolic compounds include phenolic acids, flavonoids, and anthocyanins. Phenolic acids and flavonoids are discovered in most of the plants with antioxidative effects, while anthocyanin is a member of phenolic compound isolated from red-purple colored plant.

Anthocyanins are mainly present in purple colored fruits, such as berries, blackcurrant, plum, and some indigenous fruits. *Canarium odontophyllum* (CO) fruit has high fat content with purple colored skin and high in anthocyanin. The fruit is widely cultivated in the Borneo Island, and it is an underutilized fruit in Sarawak, Malaysia. CO fruit has oily pulp with high carotenoid content. The fruit has been well described with regards to its physical properties [2] and nutritional values [3], [4].

Anthocyanins are mainly found in CO peel which consists of ∼70% of total phenolic compounds detected in the whole fruit. Major phenolic compounds and anthocyanidins were determined in the pericarp and peel of CO fruit [5]. Recently, several other major phenolic compounds and anthocyanins have been detected in the pericarp and peel of defatted CO fruit. The defatted peel of CO fruit contains high anthocyanins, also known as potential antioxidant sources [6], [7]. As compared to most of the phenolic compounds, anthocyanin possesses stronger antioxidant capacity [8]. Anthocyanidin especially delphinidin are hydrophilic [9]. However, cyanidin, as the major anthocyanidin in the peel of CO, is a less polar compound.

Anthocyanins are potential cardioprotective agent. They are known to reduce risks of several chronic diseases [10] especially cardiovascular diseases (CVD) through suppression of oxidative stress and inflammatory markers [11]. Impaired regulation of LDL is known to be caused by oxidative stress. Increased oxidized LDL in blood plasma has been shown to up-regulate CD36 expression [12], where CD36 is a critical factor in formation of atherosclerotic foam cell [13]. As antioxidant, anthocyanins are able to reduce oxidation through free radical scavenging effect, thus inhibiting expression of CD36. Dietary anthocyanins are also able to reduce LDL oxidation and oxidative damage in endothelial cells [14].

As the peel of defatted CO is rich in anthocyanin, this study sought to determine antioxidative and cardioprotective properties of anthocyanin-containing extract of defatted CO. Anti-oxidative stress effects of anthocyanin-rich extracts were evaluated using cell culture assays and LDL-oxidation method. It is also worth investigating the potential of the anthocyanin extracted from defatted CO to inhibit LDL-related oxidative stress and lipid peroxidation of human cell lines in order to identify its potential as future active pharmaceutical ingredient for cardioprotection.

## Materials and Methods

### Sample Preparation

Fresh CO fruit was purchased from the Agriculture Research Centre of Sarawak, Malaysia. Selection of the fruits and field sampling were done by the farmers and the staffs from the research centre. The fruits had been identified based on the herbarium voucher specimens (S 40073-Niah, S 64872-Kapit and S 73759-Tebedu). The fruits were transported to laboratory and stored in −20°C before sample preparation. Kernels of the fruit were manually removed from pericarp and anthocyanin-rich peel was separated from the fat-rich pericarp. Before defatting, the pericarp and peel were freeze dried using a freeze dryer (Virtis, New York, NY, USA). Lyophilized samples were ground into powder, defatted using hexane, and extracted based on an optimized extraction method. Excluding chemicals whose supplying companies have been mentioned, the remaining chemicals and reagents were purchased from Sigma-Aldrich (Selangor, Malaysia).

### Sample Extraction

Defatted CO pericarp and peel were extracted based on a response surface methodology optimized method [15]. Triplicate defatted CO samples (2.0 g) were mixed with 20 ml of 53% methanol (Fisher Scientific, Loughborough, UK). The mixture was sonicated for 1 min at high power sonication (40 kHz) using Power Sonic 405 Ultrasonicator (Hwashin Technology Co., Seoul, Korea) to increase the extraction yield. The solvent was fully removed using a rotary evaporator (Buchi, Flawil, Switzerland). The mixture was further freeze-dried to remove excessive water. The lyophilized crude extracts of defatted CO were re-dissolved with 80% methanol (Fisher Scientific, Loughborough, UK) and DMSO (Fisher Scientific, Loughborough, UK) for *in vitro* bioassays and cell culture assays, respectively. Cyanidin-3-glucoside (≥95% HPLC purity) was dissolved in 80% methanol and used as anthocyanin standard for comparison in selected *in vitro* bioassays.

### Cell Cultures

Chang liver cells (human normal cell line) (ATCC, VA, USA) and HUVEC (human umbilical vein endothelial cells) (ATCC, VA, USA) were grown in culture using 25 cm^2^ polystyrene flasks (Falcon). The Chang liver cells were maintained at 37°C in a humidified 5% CO_2_ atmosphere and cultured in RPMI medium supplemented with 10% fetal bovine serum, and 1% antibiotic, while the HUVEC were cultured in F-12K medium (ATCC, VA, USA) containing 10% fetal bovine serum (FBS), 1% antibiotic-antimycotic solution, 0.1 mg/ml heparin, and 0.03 mg/ml endothelial cell growth supplemented under an atmosphere of 5% CO_2_ at 37°C. The cells were subcultured and split 1∶4 every 4 days.

### Cell Cytotoxicity Assay

Cytotoxity assay was determined based on a modified method of Lima et al. [16]. As liver plays an important role in metabolism of nutrients, hepatotoxicity commonly occurs especially due to intake of toxic plant extract. Human liver cell lines were used to determine the cytotoxicity of defatted CO extracts. Chang liver cells were plated in 96-well culture plates at 1×10^5^ cells per well. After 24 h of cell planting, the medium was discarded and added with fresh medium containing extracts of defatted CO (0.025–1.00 mg/ml) or cyanidin-3-glucoside (C3G) (5.0–200.0 µg/ml). Cell viability was determined using a tetrazolium based MTT [3-(4,5-dimethylthiazol-2-yl) 2,5-diphenyl tetrazolium bromide] assay for triplicate analyses after 24 h of incubation.

### Cell Cytoprotective Assay

In order to determine the concentration of defatted CO extracts that protects 50% of cells death induced by the toxicant (IC_50_), two conditions were created: (i) HUVEC and Chang liver cells were induced with 2 mM *t*-BHP for 5 h, and (ii) Chang liver cells were incubated with 40% methanol for 24 h to cause significant cell death. These two cell lines were used for cytoprotective assay as the cell lines are the most commonly used human normal cell lines. Procedures from Lima et al. [16] were used for referencing.

Prevention of lactate dehydrogenase (LDH) leakage (cell death) is commonly measured in co-incubations with plant extracts that are dissolved in DMSO at 1% v/v final concentration, and the control with DMSO only. Extracts of defatted CO at several concentrations (0.025–1.0 mg/ml) were used to determine cytoprotective effect of 40% methanol and *t*-BHP-induced cell death based on MTT assay. For 40% methanol-induced cell death, the samples were dissolved in aqueous methanol. Alcohol-induced toxicity was tested using liver cells as liver is the main site for breaking down of alcohol. IC_50_ concentrations of the samples from triplicate determinations were calculated from percentages of cell viability in *t*-BHP-induced cell death of Chang liver cell.

### Colorimetric MTT (Tetrazolium) Assay

MTT (3-(4,5-dimethylthiazol-2-yl)-2,5-diphenyl tetrazolium bromide) was dissolved in phosphate-buffered saline (PBS) at 5 mg/ml and filtered to sterilize and remove small amount of insoluble residues present in some batches of MTT [17] using a modified version of Mosmann's method [18]. MTT solution was added to 96 well culture plates containing the treated cells. The plates were incubated at 37°C for 4 h. DMSO was then added to all the wells and mixed thoroughly to dissolve the dark blue crystals. After a few minutes standing at room temperature, the plates were read using a MR-96A microplate reader (Mindray, China) at test wavelength of 540 nm.

### Cellular NAD^+^ Assay

NAD^+^ assay was performed based on a method described by Geraets et al. [19] with slight modification. Chang liver cells were plated in 96-well culture plates (approximately 3×10^4^ cells/well), and the cells were cultured for 24 h before exposure to hydrogen peroxide (H_2_O_2_) (Merck Millipore, Selangor, Malaysia). H_2_O_2_ was used to induce DNA strand breaks, activate PARP-1, and deplete cellular NAD^+^ levels. To determine the optimal H_2_O_2_-concentration and incubation period for the cells, first the cells were treated with 300 µM of H_2_O_2_ for 30 min. Maximal decreases in NAD^+^ levels of Chang liver cells were found after treatment with 300 µM H_2_O_2_ for 30 min. C3G was served as positive control.

During the experiments, cells were pre-exposed to three different concentrations of defatted CO extracts [low extract concentrations for both pericarp and peel of defatted CO extract (0.025 mg/ml); IC_50_ concentration for peel and pericarp of defatted CO extract (0.153 and 0.306 mg/ml, respectively); high extract concentrations for both pericarp and peel of defatted CO extract (1.0 mg/ml)] or C3G (200 µg/ml) for 15 min, and subsequently exposed to 300 µM H_2_O_2_ for 30 min. The maximal DMSO-concentration was 0.1% (as the negative control). After the exposure, the cells were lyzed in ethanol (50 µl/well) for 10 min and stored at −80°C. During the lysis, 20 mM isonicotinic acid hydrazine was used to inhibit NAD^+^ glycohydrolase and prevent NAD^+^ hydrolysis. After thawing and lysis of the cells, 100 µl of the reaction mixture without ethanol was added to the wells. The reaction mixture in the plate contained 114 mM bicine, 4.8 mM EDTA, 0.95 mg/ml BSA, 47.6 mg/ml alcohol dehydrogenase, 1.9 mM phenazine ethosulfate, and 0.48 mM MTT. The reaction was measured spectrophotometrically at 540 nm after 15 min incubation at 37°C using a Secomam RS232 ultraviolet-visible spectrophotometer (Cedex, France). A standard curve (0–1.5 mM) was used to calculate the NAD^+^ levels in the cells. The calculated NAD^+^ levels for each experiment were the average values of triplicate measurements.

### Animal Care

Approval of the experimental protocol involving blood withdrawal from rats' tails was granted from the Animal Care and Use Committee of Faculty of Medicine and Health Sciences, Universiti Putra Malaysia (UPM), Selangor, Malaysia (Approval no.: UPM/FPSK/PADS/BR-UUH/00384). The rats (aged 8–10 weeks) were acclimatized for 2 weeks before the withdrawal of blood. A total of 10 male Sprague Dawley rats were caged individually in controlled ambient temperature of 25°C. The rats were supplied with normal chow and tap water daily. Plasma was collected by centrifugation at 1000 *g* for 10 min at room temperature and the bedding was changed every one week. Healthy rats were fed with high fat diet for 2 weeks to induce obesity by mixing beef tallow to the ground rat's chow and oven dried.

### LDL Isolation

LDL isolation was performed based on a method described by Visavadiya et al. [20] with slight modification. Blood samples were collected from the healthy fasting normal and obese adult male rats in heparinized tubes. The plasma (1.0 ml) obtained was added with 10.0 ml of heparin-citrate buffer (0.064 M sodium citrate and 50K IU/l heparin, pH 5.05) and mixed using a vortex mixer. The mixture was allowed to stand for 10 min at room temperature. Insoluble lipoprotein was precipitated by centrifugation at 1,000 *g* for 10 min. The pellet was collected and re-suspended in 1.0 ml of 0.1 M PBS (0.9% NaCl) at pH 7.4. The protein concentration of freshly prepared LDL was determined following the Lowry method [21] using bovine serum albumin for standard calibration.

### Inhibition of LDL Binding to Endothelial Cells

LDL binding study was performed based on a modified method of Dashti et al. [22]. HUVEC culture plates were pre-cooled for 30 min at 4°C. For LDL binding, the cells were incubated with 2.0 ml of fresh cold medium supplemented with 10 mM Hepes (pH 7.4), LDL solution (80 µg/ml), and defatted CO extracts (IC_50_ concentrations) to a final volume of 3.0 ml. At the end of incubation (3 h) at 4°C, the medium was removed and the cells were washed three times each with 2.0 ml of ice-cold PBS (pH 7.4), 2.0 ml of PBS containing 0.2% bovine serum albumin, and finally with 2.0 ml of PBS alone.

The amount of LDL bound to the cell surface was determined by treating the monolayers with 1.0 ml of 0.05% trypsin and incubated for 4 min at 37°C. The LDL protein released by the trypsin was measured directly using Lowry method [21]. The monolayers were scraped off from the plates with a rubber policeman after addition of 1.0 ml of PBS and sonicated for 1 min. The cells were centrifuged at 1000 *g* for 10 min at 4°C. The cell protein level was re-measured using Lowry's method. Three replicates analysis were performed for the LDL binding assays.

### Copper-Induced LDL Oxidation

Low density lipoprotein (LDL) oxidation was carried out based on a method described by Tsoukatos et al. [23] with some modifications. Pooled LDL-containing fractions obtained were suspended in PBS (1.7 ml, pH 7.4) in a final volume of 2 ml containing 80 g/l LDL protein and 4 µM copper chloride (CuCl_2_). The reacting mixture was incubated at 37°C for 3 h with and without addition of defatted CO extracts (IC_50_ concentrations) or C3G (10 µg/ml). The control analysis was performed without adding CuCl_2_ or sample extract. The oxidation was terminated by addition of EDTA (0.01%, final concentration). Oxidation product was measured using TBARS method as described by Buege and Aust [24]. Sample analysis was subjected to background subtraction and all analyses were performed in triplicate.

### Hydrogen Peroxide-Induced Hemoglobin Oxidation

Hemoglobin oxidation was performed based on a method described by Rodríguez et al. [25] with some modifications. After the second wash, the packed red blood cells obtained from both healthy fasting normal and obese rats were suspended with PBS to obtain a 5% haematocrit and pre-incubated at 37°C for 10 min in the presence of 1 mM NaN_3_ (to inhibit catalase activity). Aliquots of red blood cells (1.6 ml) were added into test tubes and incubated with 0.2 ml of 10 mM hydrogen peroxide (H_2_O_2_) with or without addition of defatted CO extracts (IC_50_ concentration) or (cyanidin) (10 µg/ml) as reference. After 60 min incubation at 37°C, the reacting mixture was placed in an ice bath for 60 s and centrifuged at 1000 *g* for 10 min at 4°C. The supernatants of triplicate measurements were used for determination of TBARS values resulting from H_2_O_2_-induced oxidation. The oxidation was terminated by addition of EDTA (0.01%, final concentration). TBARS values were measured using TBARS method described by Buege and Aust [24]. Sample analysis was subjected to background subtraction and all analyses were performed in triplicate.

### CD36 ELISA Assay

CD36 ELISA assay was performed based on a method described by Wang et al. [26]. A 100 µl of 5 µg/ml oxidized LDL was used to coat 96-well plates and incubated at 4°C for 8–12 h. The oxidized LDL-coated wells were washed with PBS buffer twice and blocked with PBS buffer containing 1% bovine serum albumin (BSA) for 1 h at 4°C, followed by washing with PBS-T (PBS buffer with 0.1% Tween-20) buffer twice. Aliquots of 10 µl crude extracts of defatted CO (IC_50_ concentration) or C3G (10 µg/ml) were added to the wells after incubated with 100 µl of 30 µg/ml CD 36 antibody at 4°C for 1 h. After incubation for 2 h, the plate was washed with PBS buffer, and then incubated with mouse monoclonal antibody against His-tag protein at a final dilution of 1∶1250 (4°C, 2 h). The wells were washed and incubated with 1∶5000 dilution of HRP-conjugated goat antimouse IgG for 1 h at 4°C. After 1 h of incubation, 100 µl of TMB/H_2_O_2_ (1∶1 [v/v]) was added to the wells and incubated for 45 min at 37°C. Finally, 100 µl of 1 M HCl was added to terminate the reaction. Absorbances of triplicate determinations were read at 450 nm using a microplate reader.

### Statistical Analysis

Data were presented as means ± standard deviations. The results were analyzed using Minitab statistical software (version 15). Analysis of variance (ANOVA) was used to determine the significant differences among the groups (p<0.05), coupled with LSD multiple comparisons.

## Results and Discussion

### Cytotoxicity and Cytoprotective Effects

Chang liver cell line is a typically normal cell line representing hepatic cells, while HUVEC is one of the endothelial cell lines commonly used for vascular studies [27]. In this study, hepatic cells (Chang liver cell line) were used to determine the effectiveness of defatted CO peel extracts in inhibiting 40% methanol-induced cell death. This model was used to mimic a chronic alcohol intake at which alcohol content of the liquor is as high as 40% volume. Methanol was used to induce cell death as the defatted CO extracts are highly soluble in methanol. In addition, 40% ethanol is not toxic enough to induce hepatic cell death.

**Figure 1:**
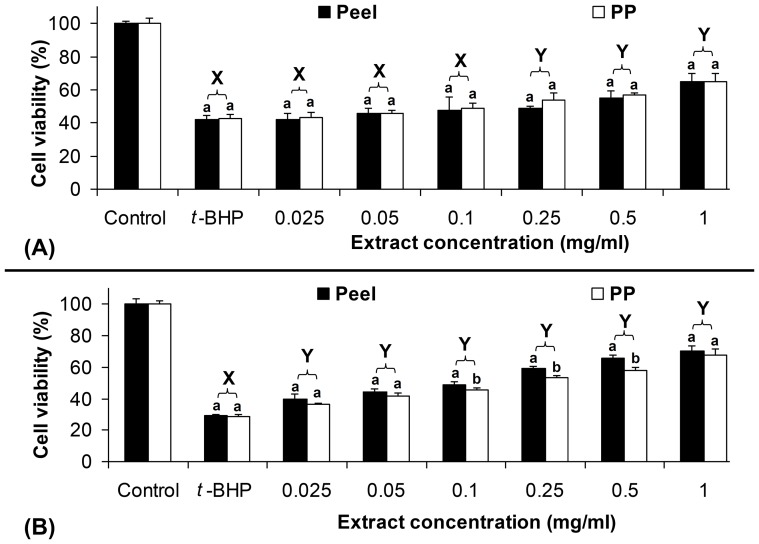
Inhibition of *t*-BHP-induced cell death by defatted CO extracts. HUVEC (A) and Chang liver cell line (B) were treated with different concentrations of defatted CO peel and pericarp (PP) extracts. Different upper case letters (X–Y) show significant differences between the extract concentrations and *t*-BHP-induced control (p≥0.05), while similar lower case letter (a or b) of the same extract concentration shows no significant difference between the peel and pericarp (PP) (p≥0.05).

Cytotoxic and cytoprotective effects of defatted CO extracts were determined using cell culture technique. Due to the high antioxidant capacity of anthocyanins in defatted CO peel [4], therefore it has the potential to exhibit good cytoprotective effect through scavenging free radicals. The results showed that the defatted CO peel extract inhibited >30% of *t*-BHP-induced cell death of HUVEC and Chang liver cell lines (Figure 1). At higher extract concentration (1.0 mg/ml), the defatted CO peel extract showed a stronger inhibitory ability compared to the treatments at lower extract concentrations (0.025–0.5 mg/ml) (Figure 1).

The extract of defatted CO peel showed lower inhibitory effect to *t*-BHP-induced cell death of HUVEC than the pericarp extract, where no significant difference in the cell viability was observed for the inhibitory effect between the extracts of defatted CO pericarp and peel. As compared to the extract of defatted CO pericarp, the peel extract had significantly higher inhibitory effect for *t*-BHP-induced cell death of Chang liver cell line at extract concentrations of 0.1–0.5 mg/ml. All concentrations of defatted CO pericarp and peel extracts used were significantly different from the non-induced and *t*-BHP-induced controls (p<0.05) except for extract concentrations of 0.025–0.1 mg/ml for HUVEC. The results also showed *t*-BHP-induced controls for Chang liver cells and HUVEC had significantly lower cell viability than the non-induced controls.

IC_50_ concentration of the defatted CO pericarp and peel extracts were calculated based on linear curves obtained from the graph of percentage of cell death versus extract concentration, where y = 28.666 x+41.23 (R^2^ = 0.8908) and y = 51.799 x+42.076 (R^2^ = 0.9128) for the defatted CO pericarp and peel extracts, respectively. The IC_50_ concentrations of the defatted CO pericarp and peel extracts were 0.306 and 0.153 mg/ml, respectively. IC_50_ concentration of defatted CO peel extract was two times lower than the pericarp indicating that similar extract concentration of defatted CO peel has twice better protective effect against oxidative damage compared to the pericarp extract. Nevertheless, protective activity of the defatted CO extracts in other biological systems might be varied due to the different extract concentrations used. In certain biological-mimicking systems, high extract concentration is needed to show high protective effect, while many other biological systems may need lower doses [28].

**Figure 2:**
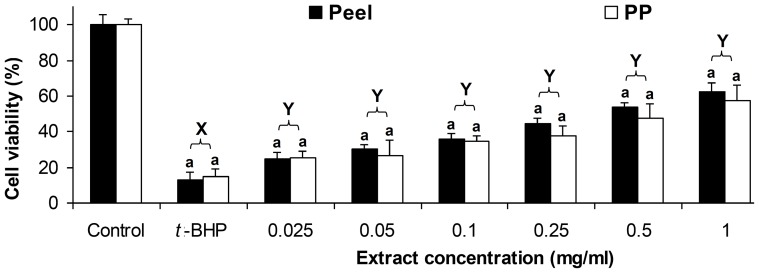
Inhibition of 40% methanol-induced cell death by defatted CO extracts. Chang liver cells were treated with different concentrations of defatted CO peel and pericarp (PP) extracts. Different upper case letters (X–Y) show significant differences between the extract concentrations and 40% methanol-induced control (p<0.05), while similar lower case letter (a) shows no significant difference between peel and pericarp (PP) (p≥0.05).

The results also showed that both defatted CO pericarp and peel extracts had equal inhibitory effect on inhibition of 40% methanol-induced cell death of Chang liver cell line (Figure 2), where no significant difference was found for the percentage of cell viability between defatted CO pericarp and peel extracts (p≥0.05). As compared to 40% methanol-induced control, both defatted CO extracts showed significant improvement in cell viability for all extract concentrations (p<0.05); while the percentages of cell viability for the non-induced control were also significantly higher than the extracts supplemented cell line.

**Figure 3:**
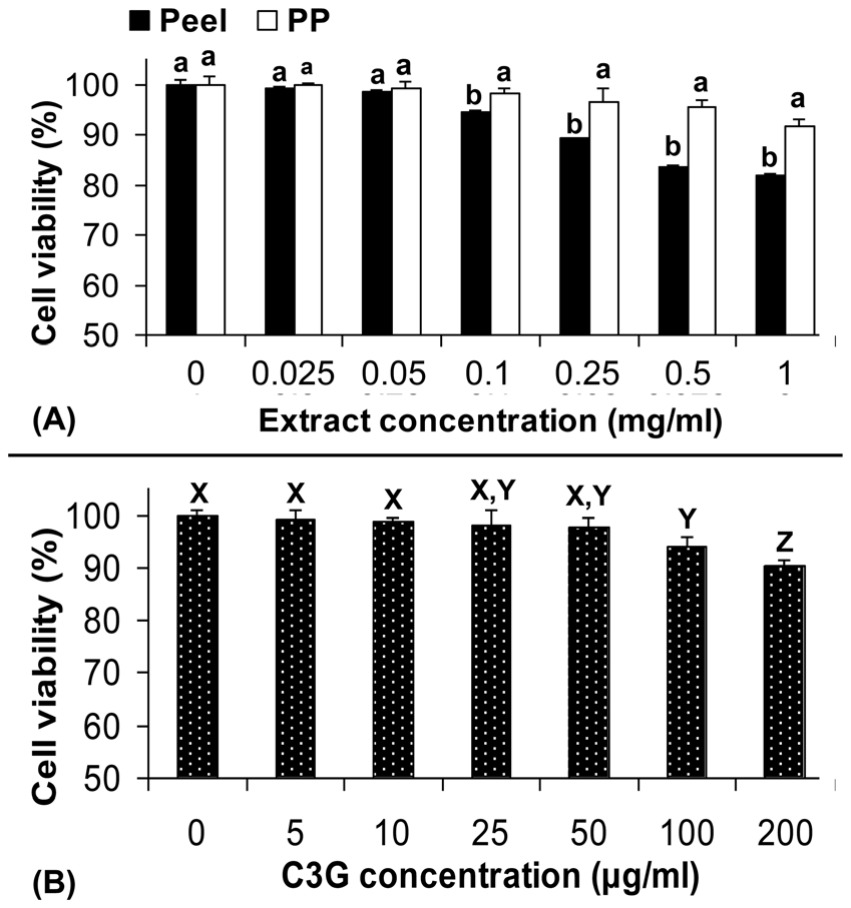
Percentages of cell viability of Chang liver cells by defatted CO extracts. Chang liver cells were treated with different concentrations of defatted CO peel and pericarp (PP) extracts (A) and cyanidin-3-glucoside (B) for comparison. Different lower case letters (a, b) show a significant difference between peel and pericarp (PP) (p<0.05), while similar upper case letters (X–Z) show no significant difference between two different extract concentrations of C3G (p≥0.05).

This study revealed that defatted CO peel extract was a potential cytoprotective agent against *t*-BHP and 40% methanol-induced cell death to HUVEC and Chang liver cell lines. The inhibitory effects of defatted CO extracts have shown a promising cardioprotection in the tested system used with no cytotoxic effect to normal cells. As shown in Figure 3, Chang liver cell line treated with 0.025–1.0 mg/ml of defatted CO pericarp and peel extracts had high percentages of cell viability. As compared to defatted CO pericarp extract, the defatted CO peel extract showed significantly lower percentage of cell viability for the extract concentrations ranged from 0.1 to 1.0 mg/ml. It shows that defatted CO pericarp extract is less toxic as compared to the peel extract. However, at low extract concentrations (0.025 and 0.05 mg/ml), both pericarp and peel extracts had equally high percentages of cell viability which was also not significantly different from the non-extract treated cell line.

Chang liver cell line treated with 5–200 µg/ml of C3G standard showed high percentages of cell viability (>90%). Similar trend was recorded for C3G (5–200 µg/ml), as the concentration decreased, cell viability increased. However, treatment with 200 µg/ml of C3G had higher percentage (>90%) of cell viability if compared to 1.0 mg/ml of the defatted CO extracts tested. As 1 mg of defatted CO peel extract contained ∼55 µg/ml of C3G, the lower cell viability of the extract used (1 mg/ml) was possibly due to the small amount of saponin found in the defatted CO peel [6].

Due to their non-toxic effect and significant reduction in *t*-BHP and 40% methanol-induced oxidative stress in hepatic as well as endothelial cells, the defatted CO pericarp and peel extracts are potential sources of pharmaceutical ingredient. The major bioactive compound in the defatted CO peel extract is C3G that reduced *t*-BHP and 40% methanol-induced cell death. Previous study reported that anthocyanin-rich extract of tea had significant protective effect against *t*-BHP-induced LDH leakage on human embryonic kidney (HEK 293) cells [29], where LDH leakage indicates apoptotic cell [30]. While H_2_O_2_-induced apoptosis to PC12 cell line (derived from a pheochromocytoma of rat adrenal medulla) was inhibited by anthocyanin-rich extract of strawberry, where the anthocyanin-rich strawberry possessed higher inhibition activity compared to other phenolic-rich fruits (orange and banana) [31]. This finding shows that anthocyanin is a stronger free radical scavenger and possesses higher inhibitory effect on oxidative damage compared to other phenolic compounds [32].

### Inhibition of Oxidative Stress

**Figure 4:**
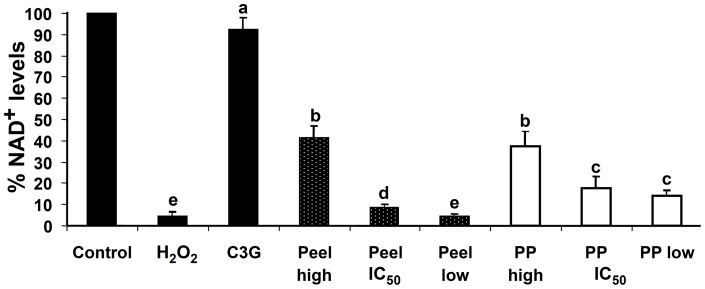
Protective effect of defatted CO extracts on depletion of NAD^+^ in H_2_O_2_-induced Chang liver cells. Values are expressed as % of control incubation (without H_2_O_2_). High, low, and IC_50_ concentrations of defatted CO peel and pericarp (PP) extracts were tested. Cyanidin-3-glucoside (C3G–200 µg/ml) was for comparison. Similar lower case letters (a–d) show no significant differences between two different extracts or between extract and H_2_O_2_/C3G (p≥0.05).

Inhibition of oxidative stress using defatted CO extracts was evaluated using cellular NAD^+^ assay and CD36 ELISA. NAD^+^ levels were measured to determine the inhibition effect of oxidative stress in H_2_O_2_-induced Chang liver cells treated by defatted CO extracts. Incubation of Chang liver cells with 300 µM H_2_O_2_ for 30 min caused more than 90% decrease in percentage of NAD^+^ compared to non H_2_O_2_-induced control. The *in vitro* accumulation of NAD^+^ was inhibited by the defatted CO pericarp and peel extracts. As shown in Figure 4, high extract concentration (1.0 mg/ml) treated to the cells significantly increased the percentage of NAD^+^ compared to low extract concentration (0.025 mg/ml).

IC_50_ concentrations of the defatted CO pericarp and peel extracts (0.306 and 0.153 mg/ml, respectively) had moderate inhibition of oxidative stress, while 200 µg/ml of C3G inhibited >90% production of NAD^+^ due to the H_2_O_2_-induced oxidative stress as compared to the non-induced control. The IC_50_ concentration of defatted CO peel extract showed higher inhibitory effect as compared to the IC_50_ concentration of the pericarp extract, and significantly different in the percentage of NAD^+^ (p<0.05) was found between the values of IC_50_ and low concentrations of the peel extract.

**Figure 5:**
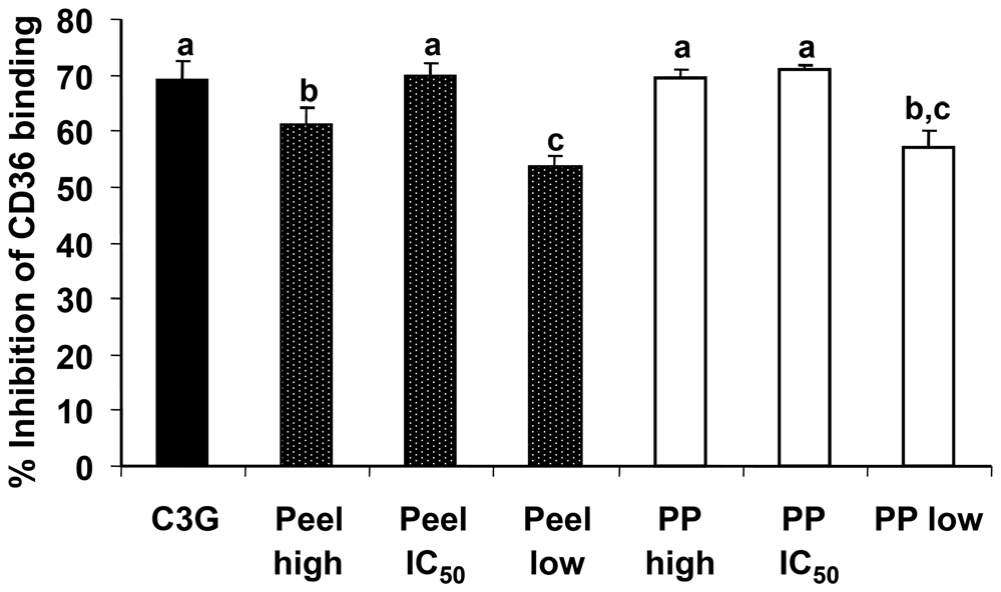
Inhibition of CD36 binding to oxidized LDL by defatted CO extracts. Values are expressed as % of inhibition. High, low and IC_50_ of concentration of defatted CO peel and pericarp (PP) extracts were tested. Cyanidin-3-glucoside (C3G–10 µg/ml) was for comparison. Similar lower case letters (a–c) show no significant differences between two different extracts or between extract and C3G (p≥0.05).

In this study, the defatted CO pericarp and peel extracts also inhibited the binding of oxidized LDL to CD36 protein. The results showed that moderate concentrations of the extract had high percentages of inhibitory effect. IC_50_ concentrations of the defatted CO pericarp and peel extracts showed the highest percentages of inhibition of oxidized LDL binding to CD36. However, at low concentration of C3G (10 µg/ml), it has similar effect as for the IC_50_ concentrations of defatted CO extracts. The high concentration (1.0 mg/ml) of defatted CO peel extract showed a significantly lower (p<0.05) percentage of inhibition if compared to its IC_50_ concentration (Figure 5). Therefore, it can be concluded that high dose of anthocyanins might somehow promote binding of oxidized LDL to CD36 receptor.

As it has previously been reported, about 80 µg/ml of total anthocyanins was detected in one milligram of defatted CO peel extract, with 55 µg/ml being C3G in one milligram of the extract [6]. It is expected that high concentration of C3G would result in a higher percentage of inhibition of oxidized LDL binding to CD36 as compared to the lower concentration. However, 10 µg/ml C3G (standard) had up to 70% inhibition activity as compared to the high extract concentration of defatted CO peel (61%). By calculation, IC_50_ concentration of the defatted CO peel (0.153 mg/ml) has concentration of C3G at 8.4 µg/ml, while the low extract concentration (0.025 mg/ml) has a calculated C3G concentration of 1.38 µg/ml. Similar with the defatted CO peel extract, calculated IC_50_ and high concentrations of the defatted CO pericarp extract were 1.9 and 6.1 µg/ml of C3G concentrations, respectively. IC_50_ concentration of the pericarp extract had the highest percentage of inhibition, but not significantly different from the percentages of inhibition for C3G (10 µg/ml), IC_50_ concentration of the defatted CO peel extract (0.153 mg/ml), and high extract concentration of the defatted CO pericarp (1.0 mg/ml).

Both low extract concentrations (0.025 mg/ml) of defatted CO pericarp and peel used in this inhibition assay resulted in less than 60% inhibition of oxidized LDL binding to CD36 receptor (Figure 5). Based on this assay, the calculated concentrations of C3G (2–10 µg/ml) in the samples and standard showed high inhibitory effect (∼70% inhibition activity). Besides C3G, other anthocyanins present in the extracts might have contributed to the high inhibition effect of oxidized LDL binding. In order to obtain an optimal level of inhibition, a series of screening tests using different sample or anthocyanin concentrations are needed to be done in future. Therefore, the inhibition assay has confirmed that anthocyanin-rich extract is a potential cardioprotective agent, where inhibition of CD36 binding to oxidized LDL potentially help to reduce the risk of CVD.

Reduction of cellular NAD^+^ is typically observed in high oxidative stress condition [33]. NAD^+^ plays an important role in metabolism through transferring electron from one molecule to the other. During oxidative damage, PARP-1 is activated followed by depletion in NAD^+^. Our previous study has shown that defatted CO peel extract highly inhibited the PARP-1 activity (unpublished data). In this study, the defatted CO peel extract has also significantly inhibited the depletion of cellular NAD^+^. Cellular oxidative damage is one of the risk factor for cardiovascular disease [34]. Oxidative damages arise from stressors such as free radicals and toxic substances are able to cause DNA damage to cells [35].

During oxidative damage, inhibition of PARP-1 by antioxidants is related to increase in expression of CD36 gene. This is supported by the fact that inhibition of PARP-1 upregulates transcription of PPARγ-target genes including CD36 [36]. Rubic et al. [37] reported that niacin (antioxidant vitamin B3) stimulated transcription of CD36 in monocytoid cells, and that CD36 was the most important scavenger receptor expressed by macrophages in uptake of oxidized lipoproteins [38]. Oxidized LDL has been shown to bind CD36 expressed in transfected cells. However, binding of the oxidized LDL to CD36 receptor inhibits clearance of apoptotic cells before they turn into necrotic lesion [39]. Calvo et al. [40] also reported CD36 has high affinity binding to oxidized LDL, and binding of CD36 to oxidized LDL is involved in atherosclerosis [41].

### Inhibitions of LDL-Binding and Lipid Peroxidation

**Figure 6:**
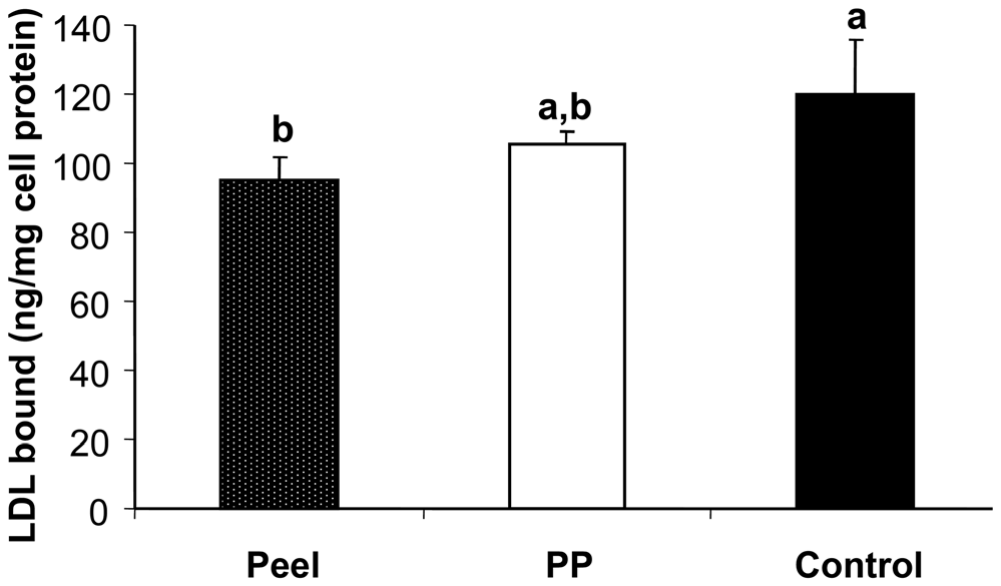
Inhibition of LDL-binding to endothelial cells by defatted CO extracts. IC_50_ concentrations of the defatted CO pericarp and pericarp (PP) extracts were used. Endothelial cells were treated with 80 µg/ml of LDL protein and incubated together with defatted CO peel and pericarp (PP) at IC_50_ extract concentration. Different lower case letters (a, b) show significant differences between two different extracts or between extract and control (p<0.05).

LDL-binding to endothelial cells is one of the risk factors for CVD [42]. The results showed that defatted CO peel extract significantly inhibited LDL-binding to endothelial cells (HUVEC) compared to control, but not the pericarp extract (Figure 6). HUVEC culture treated with the defatted CO extracts showed no significant difference in the amount of LDL bound between the pericarp and peel extracts (p≥0.05). As compared with control, defatted CO peel extract had ∼20% inhibition of LDL binding to the LDL-receptor of endothelial cells, while defatted CO pericarp extract only had ∼10% inhibition activity. Besides inhibition of oxidative stress, defatted CO peel has the ability to inhibit LDL-binding to LDL-receptor of endothelial cells.

LDL binding to LDL-receptor of endothelial cells is another potential risk for CVD. Binding of LDL to endothelial cells will eventually oxidize in high stress condition and forming oxidized LDL that is prone to induce abnormalities to the cells [43], thus leading to progression of atherosclerotic lesion [44]. Small dense LDL has increased affinity for LDL-receptor on the cell surface binding sites, and binding of the LDL to the LDL-receptor will contribute to the development of atherosclerosis [42].

**Figure 7:**
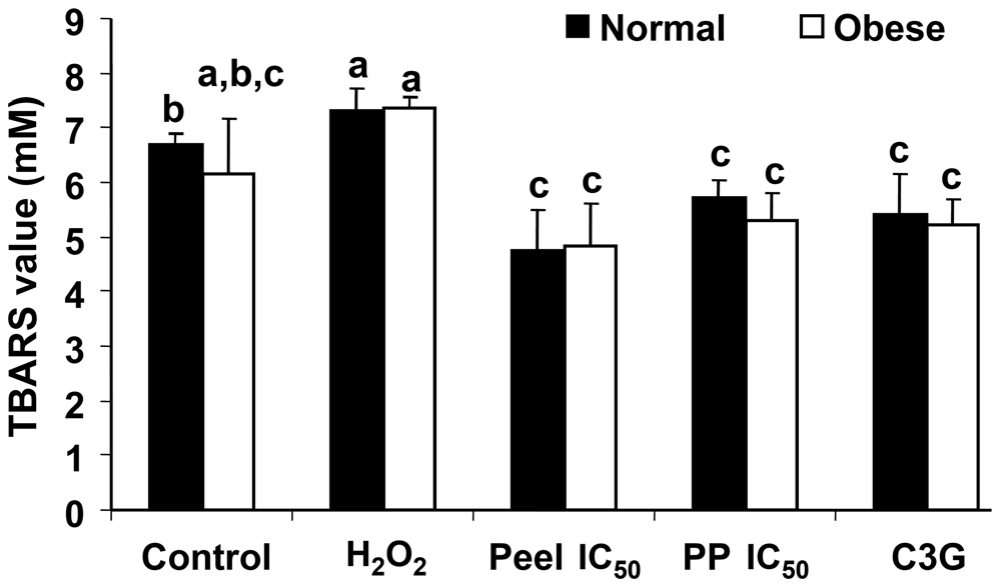
TBARS values of hemoglobin oxidation inhibition by defatted CO extracts. IC_50_ concentrations of the defatted CO pericarp and pericarp (PP) extracts were applied. Cyanidin-3-glucoside (C3G, 10 µg/ml) was for comparison. Red blood cells were obtained from a pool of blood from both normal healthy fasting and obese fasting rats (n = 5 each). No significant difference was found for the TBARS values between normal and obese rats. Different lower case letters (a–c) show significant differences between two different extracts or between extract and control/C3G for either normal or obese rats (p<0.05).

**Figure 8:**
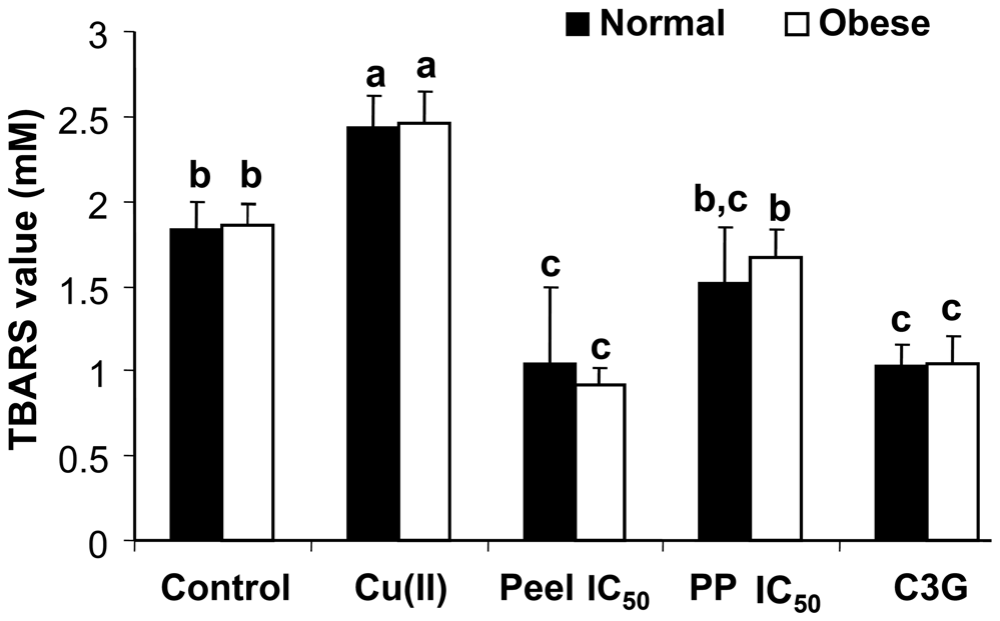
TBARS values of LDL oxidation inhibition by defatted CO extracts. IC_50_ concentrations of the defatted CO pericarp and pericarp (PP) extracts were applied. Cyanidin-3-glucoside (C3G, 10 µg/ml) was for comparison. Red blood cells were obtained from a pool of blood from both normal healthy fasting and obese fasting rats (n = 5 each). No significant difference was found for the TBARS values between normal and obese rats. Different lower case letters (a–c) show significant differences between two different extracts or between extract and control as well as C3G for either normal or obese rats (p<0.05).

In this study, inhibition of lipid peroxidation by defatted CO pericarp and peel extracts was evaluated based on hemoglobin and LDL oxidation assays. Hemoglobin and LDL obtained from normal and obese rats were used in this study. The results showed that IC_50_ concentrations of defatted CO pericarp and peel extracts significantly inhibited both hemoglobin (Figure 7) and LDL oxidations (Figure 8). In addition, no significant difference was found for TBARS values obtained from hemoglobin oxidation between normal and obese rats (Figure 7).

Defatted CO pericarp and peel extracts had TBARS values of hemoglobin oxidation comparable to the TBARS value obtained from C3G. The defatted CO extracts and C3G significantly reduced the TBARS values in hemoglobin oxidation where the blood was obtained from normal rats compared to both positive (H_2_O_2_-induced) and negative (non-induced) controls, but not for the blood obtained from obese rats. Hemoglobin from obese rats used in this assay showed no significant differences for the TBARS values between non-H_2_O_2_-induced control and treatment samples. Both H_2_O_2_-induced and non-induced controls had TBARS values significantly different from each other (p<0.05), except the blood collected from obese rats, where higher cellular oxidative stress of the obese rats increases *ex-vivo* oxidation of hemoglobin.

In LDL oxidation, LDL solution treated with defatted CO peel extract and C3G had TBARS values significantly lower than both Cu(II)-induced and non-induced controls (p<0.05) except for defatted CO pericarp extract. Although the defatted CO pericarp extract significantly inhibited Cu(II)-induced LDL oxidation, the TBARS value was not significantly lower than that of the non-induced control. Similar with the result obtained from hemoglobin oxidation, both Cu(II)-induced and non-induced controls had TBARS values significantly different from each other (p<0.05). This shows that 4 µM of copper chloride is able to drastically induce LDL oxidation. Besides having strong antioxidant capacity [6], defatted CO pericarp and peel extracts have shown protective effects against oxidative stress and lipid peroxidation. The defatted CO extracts especially the peel extract has protective effect comparable to C3G, but C3G has higher inhibitory effect of MDA formation in rat liver microsome system as compared to á-tocopherol, in which the oxidative stress was induced by Fe(III) [45].

Oxidized LDL is the root cause of cardiovascular disease as it impairs nitric oxide production and induces apoptosis in endothelial cells [46]. Inhibition of LDL oxidation by antioxidants reduces the risk of CVD. As oxidized LDL has strong affinity in binding to macrophage scavenger receptor, inhibition of LDL oxidation could have prevented the occurrence of atherosclerosis through reduction in the formation of necrotic lesions [39]. Similarly, oxidative damage of red blood cells burdens the macrophages in binding and phagocytosis processes. If coupled with increasing oxidation of LDL, the risk of atherosclerotic plaque formation becomes higher. Therefore, inhibition of hemoglobin and LDL oxidations by anthocyanins extracted from defatted CO peel should be able to reduce CVD risk.

## Conclusions

Anthocyanins extracted from defatted CO peel have cardioprotective effect. The anthocyanin-rich extract inhibited chemical-induced oxidative stress in cell cultures, reduced oxidative stress to red blood cells as well as lipoproteins, and further inhibited binding of LDL to endothelial cells. The extract also minimized binding of oxidized LDL to CD36 receptor and H_2_O_2_-induced depletion of NAD^+^ level in Chang liver cells. High extract concentration (1.0 mg/ml) of the defatted CO peel showed potentially protective effect against oxidative stress and lipid peroxidation. However, moderate concentration of the extract (IC_50_ concentration) is good for inhibition of oxidized LDL binding to CD36 receptor. The high extract concentration of defatted CO is not cytotoxic to normal liver cells as the cell viability was above 80%. Therefore, the anthocyanin-rich extracts of defatted CO are potential nutraceutical ingredient for cardioprotection. The anthocyanin-rich extracts could also be one of the pharmaceutical ingredients for prevention of CVD in the near future.
